# Seed size in mountain herbaceous plants changes with elevation in a species-specific manner

**DOI:** 10.1371/journal.pone.0199224

**Published:** 2018-06-18

**Authors:** Pawel Olejniczak, Marcin Czarnoleski, Anna Delimat, Bartosz Marek Majcher, Kamil Szczepka

**Affiliations:** 1 Institute of Nature Conservation, Polish Academy of Sciences, Kraków, Poland; 2 Institute of Environmental Sciences, Jagiellonian University, Kraków, Poland; 3 Pedagogical University of Cracow, Department of Botany, Kraków, Poland; University of Florida, UNITED STATES

## Abstract

Research devoted to investigating the relationship between elevation and seed size in alpine plants gives contradictory results. Some studies document a positive correlation between seed size and elevation, whereas in others a negative correlation is reported. We propose a novel approach to the problem by looking at the whole strategy of seed production, including seed number, and by focusing on a range of environmental variables. In the Tatra Mountains (southern Poland), we selected 73 sites at which seeds of six widely occurring mountain herbaceous species were collected. Each site was characterized by 13 parameters that included climatic and physicochemical soil variables. For each parameter, residuals from a linear regression against elevation were calculated and the residuals were used in a factor analysis. The obtained factors, together with elevation, were used as independent variables in a multiple regression analysis. Elevation affected seed size in four species: in two species the correlation was positive, and in two others it was negative. In three species seed number was related to elevation, and the correlation was negative in all cases. Our results indicate that elevation-dependence of seed production is specific to the species and reflects different resource allocation strategies. Diverse correlations of plant characteristics with elevation may also result from area-specific patterns, because different mountain ranges may exhibit different correlations between elevation and environmental factors. Only by attaining a reproductive allocation perspective and thorough assessment of environmental factors, a full understanding of elevational variation in seed size is possible.

## Introduction

Seed size varies dramatically between plant species (e.g. [1–2]), and this variance is convincingly explained by the effects of different ecological parameters [2–3]. In contrast, differences in seed size between individuals of the same species are relatively less frequently studied. Although seed size should remain under the direct control of natural selection within populations [4], we do not fully understand how environmental factors shape the intra-specific variance in seed size. Given the framework of an optimal resource allocation theory, seed size defines an investment of a parental plant into a single offspring, which affects the survival of the plant [5–6], as well as its growth and future size [7–8]. At the same time, an adaptive size of seeds has to be seen from the perspective of the size vs number of offspring continuum [9]. The classical model in this area proposed by Smith and Fretwell [10] not only predicts an optimum offspring size, but it also explicitly considers that from a limited amount of resources a greater number of offspring can be produced only at the expense of their size. Later theories considering the trade-off between seed size and seed number [11–12] predicted that an evolutionarily stable solution for a plant is not the production of seeds with a single size, but the range of seed sizes that increases with the amount of available resources [11].

Indisputably, the advantage of small seeds is that they can be produced in large numbers, which directly increases the number of gene copies that are likely to be passed on to next generations. Small seeds can also persist longer in seed banks [13], germinate faster [14], and are expected to suffer lower seed predation [15–16]. These advantages should inevitably lead to the production of the smallest viable seeds, unless a small seed has some fitness disadvantage. Indeed, many empirical studies show a positive link between seed size and plant fitness. Having more storage, a bigger seed has a better chance to establish in a habitat [17] or to tolerate hazards [18]. Larger seeds produce bigger seedlings, which prepares them for competition with already established plants [19] or speeds up the attainment of life stages less vulnerable to herbivory [20]. Clearly, to properly address the fitness consequences of seed size, we must consider a compromise between costs and benefits in a trade-off between seed size and seed number.

We conducted a field study in the highest range of the Carpathian Mountains to explore elevational variation in seed size and seed number in six perennial herbs. Both a positive and negative correlation between seed size and elevation can be predicted on theoretical grounds, and there is evidence to support either pattern. For example, Boulli et al. [21] and Pluess et al. [22] observed an increase in seed size with elevation in Morocco and in the Alps, whereas Bu et al. [23] and Guo et al. [24] observed a reverse pattern in Tibet. Interestingly, one of the most recent and comprehensive studies [25] reported that the relationship between seed mass and elevation was positive within species, but negative across species. The authors evoke the ‘stress-tolerance’ mechanism to predict that higher elevations promote larger seeds because their greater size ensures successful recruitment in high-alpine stressful environments. According to this hypothesis, a positive correlation between seed mass and elevation is predicted. In contrast, the ‘energy-constraint’ hypothesis points to a direct effect of deteriorating conditions at higher elevations: lower temperatures, shorter growing season and other adverse factors, force plants to invest less resources to seed production, which results in smaller seeds and a smaller number of seeds at higher elevations. To increase the ecological relevance of our study, along with data on elevation we also collected data on thermal and soil conditions directly in local microhabitats in which we studied individual plants. Our high-resolution data on temperatures collected over three years provided us with access to unique information on the local heterogeneity in thermal conditions of the studied plants. This way, we were able to link the variance in seed size and seed number not only to elevation and to local environmental variance at given elevations, but also to recognize the effects of particular environmental factors, such as a season length and access to nutrients. By measuring the size of individual seeds together with the total number of seeds produced by individual plants, we were also able to address questions about a trade-off between seed size and seed number and about environmental effects on the variance in seed size.

## Materials and methods

### Study sites and plant species

The study was conducted over four years (2011–2014) in the Tatra Mountains in southern Poland (49^o^10’– 49^o^16’ N, 19^o^42’– 20^o^06’ E). The Tatras form the highest range of the Carpathian Mountains and they are the only alpine-like mountain range between the Alps and the Caucasus. The elevation range in the Polish Tatras is between ca. 900 and 2,500 m, and it traverses through a series of mountain vegetation zones, from a lower montane forest up to a subnival zone. Within the area of ca. 150 km^2^, we defined four elevational transects with 73 study sites in total, encompassing different topographic, geological and geomorphologic features. A major criterion for site selection was the presence of several individual plants of at least one of our study species. The study species belong to six herbaceous perennial plants, and they were a priori chosen based on their known ubiquity at different elevations in the Tatras (Table 1). None of them is endangered or protected. All the study species are insect-pollinated, and in Central Europe, they occur mostly in mountain areas.

**Table 1:** List of studied species.

Species name	Family	Dispersal mode	Elevation (m)	
			Lower limit	Upper limit
*Primula elatior*	Primulaceae	Autochory	900	2200
*Soldanella carpatica*	Primulaceae	Autochory	900	2400
*Geum montanum*	Rosaceae	Anemochory	1300	2400
*Homogyne alpina*	Asteraceae	Anemochory	900	2400
*Leucanthemum waldsteinii*	Asteraceae	Anemochory	900	1900
*Senecio subalpinus*	Asteraceae	Anemochory	900	2200

Studied plants belong to six species, representatives of three families.

### Procedures at study sites

In autumn 2011, at a central point of each study site, one or two data loggers (EL-USB-2, Lascar Electronics Ltd.) were placed 5 cm below the soil surface. Temperature to the nearest 0.5°C was recorded every hour until summer 2014, which allowed for collecting high-resolution data on thermal conditions in local habitats from which we were harvesting seeds. At each study site, the elevation, aspect of a slope, and local topographic conditions were recorded. The aspect of slope was expressed as a sun exposure index–a convex angle measured between the north and the direction the slope faces. The maximum value of 180° was assigned to south-facing slopes, indicating the highest level of sun exposure given the latitude of the study area. The minimum value of 0° was assigned to north-facing slopes, which should experience the lowest sun exposure at these latitudes. On the basis of local topography, a snow persistence index was defined as a number on a four-step ordinal scale, with the value 0 assigned to the most convex sites and value 3 to the most concave sites, which should be characterized by the highest tendency for snow retention. For logistical reasons, the seed collection was split into two years. In 2012, we collected seeds of *Primula elatior*, *Homogyne alpina*, *Senecio subalpinus* and *Leucanthemum waldsteinii*; in 2013, we collected seeds of *Soldanella carpatica* and *Geum montanum*. The study sites were monitored many times within each season to assure that fully developed seeds were collected and to minimize the incidences of seed loss associated with natural dispersal. The target species usually form well distinguishable plants. All seeds from the shoots of an independent, distinguishable plant were collected. In cases when collecting the whole seed set from the plant was questionable due to seed predation, mechanical damage or other reasons, the plant was disregarded.

In September 2014, a volume of a half-liter soil sample was collected at each study site. This volume comprised five topsoil (5–10 cm) sub-samples taken within a distance of two meters from the central point of each site. The soil samples were kept in a freezer (-20°C) until their physicochemical properties were analyzed. The field work was confined to Tatra National Park. All field procedures were conducted according to the permission obtained from the Ministry of the Environment of the Republic of Poland.

### Assessing seed characteristics

All seeds were room-temperature dried and deprived of dispersal structures, e.g., pappus. The seeds from every parental plant were collected and scattered over a 210 x 290 mm contrasting background sheet, ensuring that their outlines did not overlap. Each sheet was printed with a 2 x 2 cm black square, which was further used to calibrate the measurements of the seeds. The sheets with seeds were photographed with a digital camera (Canon 600D, f = 50 mm) with a ring flash lamp, mounted on a regulated stand. The digital images were analyzed with a modified automated method of Schramm et al. [26]. This procedure automatically recognized seeds from artifacts, counted seeds and measured the area of their vertical projection. The mean seed area of a photographed seed set was used as an estimate of the mean seed size produced by a plant. A coefficient of variance (CV) of seed size per seed set was used as a measure of seed size heterogeneity.

Given the need to assess the size of individual seeds and their total number, we used seed area instead of seed mass as a measure of seed size, which we further analyzed to test hypotheses. To validate the use of seed area, we pooled seeds of each plant, weighed them to the nearest 0.1 mg, and checked how well between-plant differences in seed mass corresponded to differences in seed area. Using the least square nonlinear method, we fitted a power function y = a x^b^ to the data on the total seed projection area per plant (x) and the total seed mass per plant (y), separately for each plant species. For all species, we found a strong correspondence between our measure of seed size and the mass of seeds with the coefficient of determination (r^2^) of these relationships ranging from 0.845 to as high as 0.968: *P*. *elatior*: y = 3.79 x ^0.88^, n = 264, r^2^ = 0.920; *S*. *carpatica*: y = 3.25 x ^0.88^, n = 491, r^2^ = 0.878; *G*. *montanum*: y = 7.30 x ^0.73^, n = 283, r^2^ = 0.845; *H*. *alpina*: y = 3.09 x ^0.87^, n = 136, r^2^ = 0.933; *L*. *waldsteinii*: y = 3.07 x ^0.99^, n = 299, r^2^ = 0.968; *S*. *subalpinus*: y = 6.73 x ^0.87^, n = 246, r^2^ = 0.918.

### Soil analysis

Soil samples were sieved (4 mm mesh) and divided into three parts. The first part was dried (105°C, 8 h) and used to assess organic matter content, pH, and conductivity. Organic matter content was determined with a loss-on-ignition method by burning a dried sample (ca. 2 g) in an electric furnace (550°C, 2 h). A pH meter with a glass electrode was used to assess the pH of the soil in water and in KCl solution. pH was measured in 10 ml solution of soil, which was prepared by dispersing soil samples (4 g each) in a solvent (H_2_O or 1M KCl solution). Electric conductivity was measured in the water solution prepared as for measuring the pH in water. The second part of the original soil sample served to prepare a solution (5 g of fresh sample filled to 100 ml with 2M KCl solution) for the assessment of nitrate and nitrite concentration. The concentration of these compounds was determined using a flow injection analyzer. The third part of every sample was ground in a planetary micro mill (Pulverisette, Fritsch) and dried (105°C, 24 h) for the assessment of Ca, Mg, K and P concentrations. Such prepared samples (0.5 g, measured with 0.0001 g accuracy) were mixed with 10 ml of nitric and perchloric acids mixture (4:1, 65% HNO_3_:60% HClO_4_) and mineralized. The contents of Ca, Mg and K in these samples were measured by atomic absorption spectrometry (Perkin Elmer Analyst 200). The concentration of phosphates was determined with a flow injection analyzer. All the above measurements were repeated twice, and the average was used in further analyses.

### Climate parameters

Recordings of temperature from the data loggers were used to calculate the mean temperature in the warmest period of summer and the length of winter. All recorded temperatures between July 11, 2012 and August 10, 2012 (31 d, 744 recordings) were averaged, and the obtained value was used as a ‘peak summer mean temperature’ parameter. Winter was defined as a period in which low temperature and snow cover hinder plant growth. Our observations indicated that the presence of snow cover corresponds with a cessation of daily temperature fluctuations. Thus, we defined winter as a period with a daily amplitude lower than 1°C and maximum daily temperature lower than 3°C. The length of winter for each site was calculated as the average number of winter days over three years.

### Statistical analysis

Prior to the analysis, each study location was characterized by the species-specific mean values of seed size, seed number and the coefficient of variance in seed size, and a set of physicochemical parameters of a local environment: soil parameters and climate parameters (winter duration, snow cover persistence index, peak summer mean temperature, sun exposure index). To examine elevational gradients in environmental conditions and separate this effect from the effects of local heterogeneity of environmental conditions, we fitted ordinary least-square regressions to data on the physicochemical parameters and elevation. Residuals of these regressions served as measures of elevation-independent variation in the environment. To integrate information on elevation-independent environmental variance, we performed a Principal Component Analysis (PCA). The PCA extracted four principal components, which were further rotated in a Factor Analysis (FA) with the Varimax method, which helped to convert our information on many environmental parameters to a smaller number of easy-to-interpret variables (factors). In a further analysis, we used elevation as a proxy of environmental parameters that co-varied with elevation, and scores of the extracted factors as an index of environmental conditions that changed independently of elevation. Exploring the links between plant characteristics and environmental conditions, we performed two types of correlative analyses for each species. First, we analyzed simple regressions between elevation and mean seed size, mean seed number, and mean CV of seed size. Then, we performed a multiple regression with elevation and scores of factors as multiple independent variables. The latter analysis tested the independent effects of the elevational gradients in the environment and the local environmental heterogeneity on the characteristics of seed production. All statistical analyses employed SPSS ver. 24.0 [27].

## Results

As shown in Table 2, sites at higher elevations had soil with lower pH and conductivity, and lower amounts of calcium, magnesium and nitrates and nitrites, and they were characterized by longer winters with prolonged periods of snow cover, followed by short and cool summers. For example, plants in our lowest study location (917 m) experienced on average 132.7 d of winters, whereas in our highest location (2139 m) winters were 76% longer (232.9 d). Even at a given elevation, winter lengths varied over 64 d between locations.

**Table 2:** Results of regression analysis.

Environmental parameter	Regression slope	p
Sun exposure index	0.004	0.83
Snow cover persistence index	**0.002**	0.0001
Peak summer mean temperature	**-0.003**	0.0001
Length of winter	**0.082**	0.0001
Ca^+2^ in soi	**-37.892**	0.0006
Mg^+2^ in soil	**-19.029**	0.0005
Soil pH	**-0.001**	0.0002
Soil pH in KCl solution	**-0.001**	0.0005
K^+^ in soil	-0.533	0.76
Phosphates in soil	0.221	0.17
Soil conductivity	**-0.142**	0.004
Nitrates and Nitrites in soil	**-0.023**	0.03
Organic matter in soil	-0.001	0.63

Slopes of regressions are shown describing the relationships between physicochemical conditions in soil and climatic parameters against elevation. Values significant at p < 0.05 are boldfaced.

Our factor analysis of the elevation-independent variance in physicochemical parameters (Table 3) resulted in four major factors (F1–F4) that together explained ca. 70% of variance in the data. The amount of calcium and magnesium and our two measures of pH loaded positively on the first factor, indicating that higher scores of F1 correspond to alkaline microhabitats rich in calcium and magnesium. The second factor was mainly formed by the positive effects of soil pH and conductivity and the amount of potassium and phosphates; therefore, higher scores of F2 indicate alkaline habitats rich in free ions such as potassium and phosphates. The third factor corresponds mainly to differences in winter lengths–higher scores of F3 indicate locations with low exposure to sun, long winters, a high index of snow persistence, and low peak summer mean temperatures. The fourth factor corresponds to the access to organic-related nutrients. Higher scores of F4 indicate microhabitats with higher soil conductivity, rich in nitrates, nitrites, and organic matter.

**Table 3:** Results of factor analysis.

Environmental parameter	Factors			
	F1	F2	F3	F4
Sun exposure index	0.218	- 0.080	**- 0.585**	0.038
Snow cover persistence index	0.067	0.210	**0.772**	- 0.097
Peak summer mean temperature	- 0.229	- 0.152	**- 0.663**	- 0.063
Length of winter	- 0.177	- 0.251	**0.788**	- 0.226
Ca^+2^ in soil	**0.979**	- 0.062	- 0.026	- 0.005
Mg^+2^ in soil	**0.967**	0.007	- 0.045	- 0.035
Soil pH	**0.584**	**0.754**	0.004	0.070
Soil pH in KCl solution	**0.622**	**0.720**	- 0.027	0.049
K^+^ in soil	- 0.049	**0.685**	0.206	0.278
Phosphates in soil	- 0.189	**0.849**	0.097	- 0.145
Soil conductivity	0.009	**0.577**	0.053	**0.535**
Nitrates and Nitrites in soil	- 0.081	0.169	- 0.171	**0.810**
Organic matter in soil	0.050	- 0.053	- 0.057	**0.680**
Amount of explained variance (%)	21.6	21.4	16.1	12.1

Factors have been defined as a result of Varimax rotation of four principal components. The loadings for each environmental parameter assessed at all localities are given. In the analysis, residuals of the parameters used were calculated as deviations from the linear regression of the given parameter against elevation (see Table 2). Loadings with absolute value greater than 0.5 are boldfaced.

**Fig 1:**
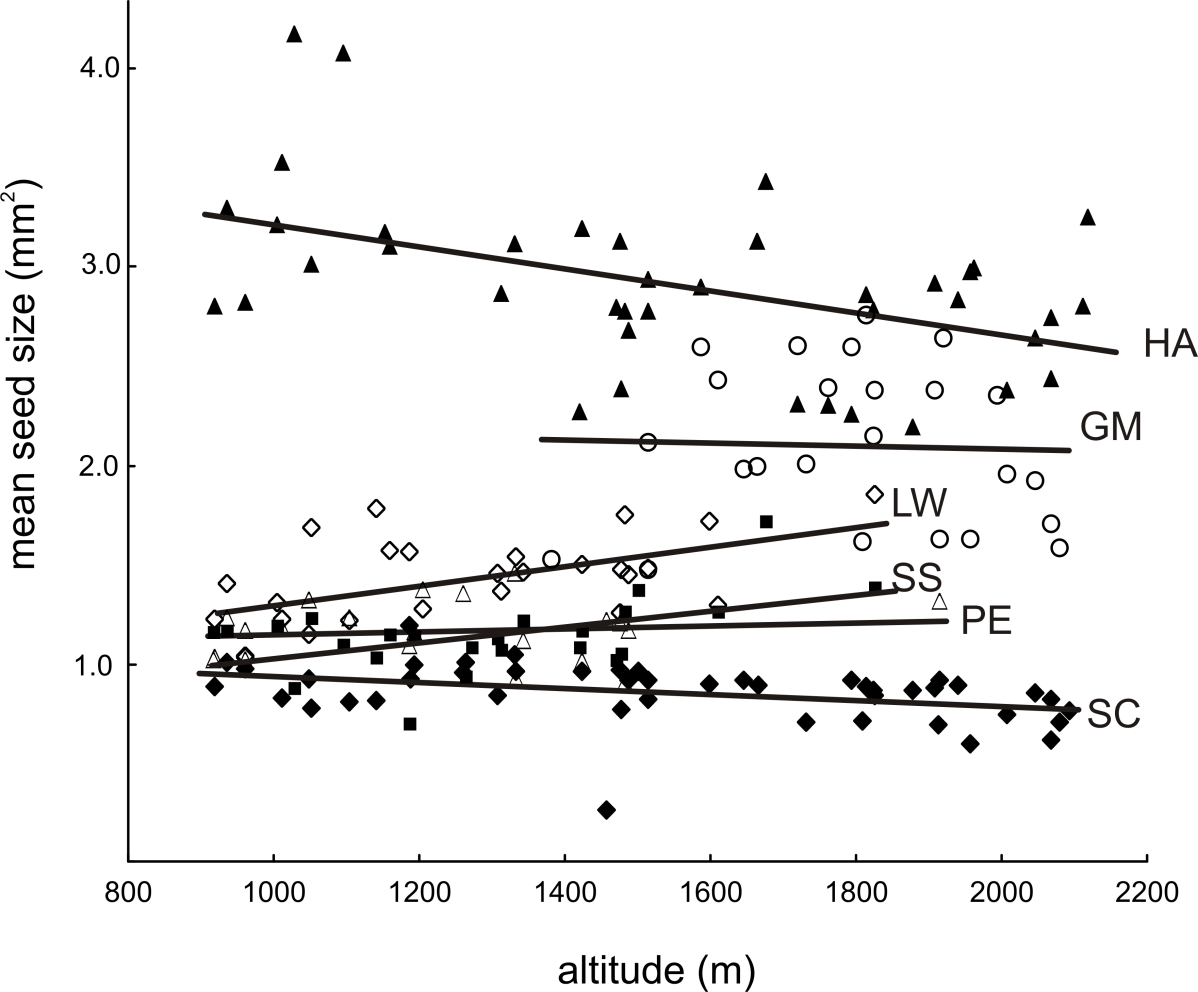
Relationship between seed size and elevation. Depending on a plant species, a mean size of seeds either increased, decreased, or remained uncorrelated with elevation. The lines represent least square regressions fitted to data on mean seed size in the study sites. PE–*Primula elatior*, SC–*Soldanella carpatica*, GM–*Geum montanum*, HA–*Homogyne alpina*, LW–*Leucanthemum waldsteinii*, SS–*Senecio subalpinus*.

If analyzed alone, elevation correlated with mean seed size and seed number, but the nature of this dependence varied between plant species (Fig 1, Table 4). The mean seed size increased with elevation in *Leucanthemum waldsteinii* and *Senecio subalpinus*, but decreased with elevation in *Homogyne alpina* and *Soldanella carpatica*. Seed size was independent of elevation in *Geum montanum* and *Primula elatior*. The production of seeds was unrelated to elevation in most species, except in *P*. *elatior* that produced fewer seeds at higher elevations. In all the species, the variance in seed size produced by individual plants was unrelated to elevation. The analysis of multiple regression with elevation as one predictor, and the scores of the factors (F1–F4) as the other predictors (Table 5), confirmed the main effects of elevation, but also revealed additional patterns. In particular, we found that seed number decreased with elevation in half of the species, namely *S*. *subalpinus*, *G*. *montanum*, and *P*. *elatior*; in the remaining species, the number of seeds remained independent of elevation. Moreover, a negative correlation between mean seed size in *S*. *subalipinus* and F1 scores indicates that at a given elevation, individuals of *S*. *subalpinus* from alkaline microhabitats rich in calcium and magnesium had smaller seeds, but the number of seeds in this species was unaffected by F1. In contrast, alkaline microhabitats rich in calcium and magnesium caused *G*. *montanum* and *S*. *carpatica* to produce more seeds with no change in mean seed size, as indicated by a positive correlation between seed number and F1 in these species, with no effect of these factors on seed size. Finally, in *G*. *montanum*, scores of F4 correlated positively with seed number, indicating that plants from microhabitats rich in organic matter and nitrogen produced more seeds. We found no indication that a heterogeneity in winter length at a given elevation had a significant impact on any characteristics of seed production in the studied plants.

**Table 4:** Correlations between seed characteristics and elevation in the studied plant species.

Species	Feature	N	r	p
*Homogyne alpina*	Seed number	40	0.1590	0.3270
	Mean seed size	40	**0.4800**	0.0017
	CV of seedsize	40	0.0108	0.9470
*Leucanthemum waldsteinii*	Seed number	26	0.2118	0.2988
	Mean seed size	26	**0.5479**	0.0037
	CV of seed size	26	0.0715	0.7283
*Senecio subalpinus*	Seed number	24	- 0.3526	0.0910
	Mean seed size	24	**0.4924**	0.0144
	CV of seedsize	24	0.2142	0.3148
*Geum montanum*	Seed number	24	- 0.2331	0.2728
	Mean seed size	24	0.0360	0.8673
	CV of seed size	24	0.3781	0.0684
*Primula elatior*	Seed number	21	**- 0.5373**	0.0119
	Mean seed size	21	0.1270	0.5832
	CV of seed size	21	0.2808	0.2175
*Soldanella carpatica*	Seed number	45	- 0.1113	0.4666
	Mean seed size	45	**0.3794**	0.0101
	CV of seed size	45	0.0511	0.7415

Correlation coefficients significant at p < 0.05 are boldfaced.

**Table 5:** Results of multiple regression with seed characteristics as a dependent variable, and elevation with factors as independent variables.

Species	Dependent variable	Independent variable	Partial correlation coefficient	p
*Homogyne alpina*	Seed number	Elevation	0.174	0.311
		F1	0.132	0.454
		F2	0.005	0.976
		F3	- 0.146	0.389
		F4	0.128	0.474
	Mean seed size	Elevation	**- 0.499**	0.002
		F1	- 0.063	0.680
		F2	- 0.034	0.831
		F3	- 0.212	0.157
		F4	0.136	0.383
*Leucanthemum waldsteinii*	Seed number	Elevation	0.286	0.231
		F1	- 0.101	0.644
		F2	- 0.054	0.814
		F3	0.247	0.291
		F4	- 0.139	0.538
	Mean seed size	Elevation	**0.654**	0.002
		F1	- 0.137	0.422
		F2	- 0.303	0.101
		F3	0.316	0.089
		F4	0.052	0.766
*Senecio subalpinus*	Seed number	Elevation	**- 0.508**	0.018
		F1	0.333	0.110
		F2	0.274	0.194
		F3	- 0.216	0.300
		F4	0.104	0.598
	Mean seed size	Elevation	**0.499**	0.012
		F1	**- 0.398**	0.041
		F2	0.183	0.337
		F3	- 0.122	0.517
		F4	0.113	0.531
*Geum montanum*	Seed number	Elevation	**- 0.873**	0.000
		F1	**0.865**	0.008
		F2	- 0.051	0.826
		F3	0.026	0.857
		F4	**0.479**	0.003
	Mean seed size	Elevation	- 0.531	0.087
		F1	0.841	0.067
		F2	- 0.351	0.311
		F3	- 0.339	0.123
		F4	0.275	0.196
*Primula elatior*	Seed number	Elevation	**- 0.602**	0.015
		F1	- 0.285	0.341
		F2	- 0.094	0.755
		F3	0.073	0.727
		F4	0.161	0.479
	Mean seed size	Elevation	0.077	0.753
		F1	0.462	0.168
		F2	0.644	0.066
		F3	- 0.052	0.821
		F4	- 0.033	0.893
*Soldanella carpatica*	Seed number	Elevation	- 0.023	0.880
		F1	**0.328**	0.040
		F2	- 0.052	0.738
		F3	- 0.148	0.337
		F4	0.039	0.797
	Mean seed size	Elevation	**- 0.323**	0.033
		F1	0.246	0.104
		F2	- 0.084	0.575
		F3	- 0.022	0.881
		F4	0.095	0.511

Partial correlation coefficients significant at p < 0.05 are boldfaced.

## Discussion

An elevational gradient in physio-chemical parameters is the most pervasive element of the mountainous environments [28]. Unsurprisingly, we found that either seed size or seed number was linked to elevation in every plant species that we studied. Nevertheless, our results demonstrate that the plants occupied a wide gradient of environmental conditions that were not directly linked to elevation. For example, our estimated length of winters increased by 100 d over the range of studied elevations, but at a given elevation locations still varied in winter length by as much as 64 d due to specific characteristics of a local microhabitat.

Generally, we found two distinct patterns in the elevation-dependence of seed production. In species in which seed number was independent of elevation (*Homogyne alpina*, *Leucanthemum waldsteinii*, *Senecio subalpinus* and *Soldanella carpatica*), we observed either an increase (*L*. *waldsteinii* and *S*. *subalpinus*) or a decrease (*H*. *alpina* and *S*. *carpatica*) in seed size with elevation. In contrast, in species in which seed size was independent of elevation, seed number decreased with elevation (*Geum montanum* and *Primula elatior*). These patterns suggest different resource allocation strategies. *L*. *waldsteinii* and *S*. *subalpinus* were the only plants that increased their total investment in seed production with elevation, which suggests that their capacity to produce seeds was not directly limited by environmental correlates of elevation. By directing more resources to seed production with elevation, *L*. *waldsteinii* and *S*. *subalpinus* increased the level of provisioning of single seeds, instead of increasing the number of seeds. In other words, they sacrificed their fertility for the quality of seeds. Consequently, despite an increased total investment in seed production, high-elevation individuals of *L*. *waldsteinii* and *S*. *subalpinus* produced as many seeds as their low-elevation conspecifics. In contrast, the remaining four species (*H*. *alpina*, *S*. *carpatica*, *G*. *montanum* and *P*. *elatior*) reduced their total investment in seed production with elevation, suggesting a direct limitation of their physiological performance with elevation. In fact, *H*. *alpina*, *S*. *carpatica*, *G*. *montanum* and *P*. *elatior* reached higher elevations compared to *L*. *waldsteinii* and *S*. *subalpinus*, where they were likely to face much more severe conditions, limiting the capacity to invest in seed production. Importantly, as elevation was increasing, one group of species prioritized seed number at the expense of seed size (*H*. *alpina* and *S*. *carpatica*), while the other species prioritized seed size at the expense of seed number (*G*. *montanum* and *P*. *elatior*).

Overall, these results suggest that all the studied species faced a trade-off between seed number and seed size, but each species conformed to a different allocation strategy. In the face of elevation-related shrinkage of summer and deterioration of thermal conditions during this period, a visible strategy was preserving (*G*. *montanum* and *P*. *elatior*) or even increasing (*L*. *waldsteinii* and *S*. *subalpinus*) seed size. Perhaps, in these species, seed size might be critical for the successful establishment of seedlings. If sites colonized by dispersing seeds are typically suitable as potential habitats, then the establishment of arriving seeds should depend on their ability to cope with competitors, grazers and time pressure of a vegetative period. This should select such plants for the production of larger seeds, even at the expense of their number. Arguably, *G*. *montanum*, *P*. *elatior*, *L*. *waldsteinii* and *S*. *subalpinus* follow such a strategy, but we can only speculate on this scenario. In contrast, if most sites encountered by dispersing seeds are not promising as potential habitats, then plants may decrease seed size but increase seed number. It is likely that such a strategy was employed by *H*. *alpina* and *S*. *carpatica*.

Earlier empirical studies aimed at examining the correlations between elevation and seed size have produced conflicting results [21–24]. An attempt to reconcile this inconsistency was proposed by Qi et al. [25]. On the basis of an extensive comparative study conducted in the Tibetan plateau, the authors concluded that seed size correlated positively with elevation at a within-species scale, but at a between-species level this correlation was negative. Interestingly, a discrepancy between inter- and intra-specific seed size correlations with elevation was also found by Pluess et al. [22], but here alpine species tended to have larger seeds than their lowland counterparts, whereas within species correlations between seed size and elevation were more likely to be negative. The authors argue that the positive relation between seed size and elevation may not be generally observed because of phylogenetic constraints or high gene flow among populations. The results obtained in our study document that both positive and negative elevation-seed size correlations occur within species. Our findings support the conclusions of an earlier study on *Rhododendron* species [29] in which intra-specific correlations between seed mass and elevation were found to vary between species. Interestingly, single environmental gradients, including elevation, were often reported to be highly complex and their effects on plant functional traits to be species-specific (e.g. [30–32]). This suggests that we should not expect a single universal pattern in the effects of external factors on plant characteristics.

The fact that different seed size-elevation correlations were revealed in different studies may be explained in two ways. Firstly, research in this field focuses on different plant species. These plants may have evolved specific adaptation to a mountainous environment, and therefore, we can observe a whole range of responses of their seed production strategy to environmental conditions that change along with elevation. Secondly, particular mountain ranges may differ in the nature of elevation-dependence of environmental factors, such as pH in soil, concentration of minerals or organic matter in soil, etc. If the seed size of a given species depends on soil characteristics, then studies conducted in different mountains may reveal different seed size correlations with elevation. In fact, the physicochemical parameters of soil were found to be differently related to elevation [33–35]. Even the neighboring mountain ranges may exhibit different elevation-soil nutrient characteristics, e.g., in Slovensky Raj in the Western Carpathians, as close as 40 km from our study sites [36], elevational gradients of soil nutrients differ substantially from what we observed in the Tatra Mountains.

Plants at higher elevations are more likely to be limited by soil nutrients deficiency and by the length of the growing season. Thus, in general, the amount of resources available for seed production is likely to decrease with elevation. These conditions are expected to favor seeds with more variable size [11], serving as a bet-hedging strategy for a plant. Against this expectation, we did not find a link between elevation and a coefficient of variance in seed size in any of our species. Even though game theory models predict certain evolutionary stable seed size variation and its dependence from external conditions [11–12], some empirical studies contradict their predictions [37], and so do our results.

Elevation, the most widely recognized critical factor characterizing mountain environments, is bound to affect seed size, the principal component of plant reproductive strategy. However, as shown by our results, the way elevation affects seed size is far from being universal. One reason for this variance could be that elevation is not a simple environmental factor that directly impact plants, but a complex variable with many components. These variable components can be responsible for the opposite elevational gradients in seed sizes in different mountain ranges, but they may remain unrecognized, unless directly assessed. On the other hand, particular plant species may react differently to shifting environmental conditions, and the partitioning of reproductive resources between many smaller seeds or few larger ones may bring different fitness consequences, depending on other features of plant strategy and local environment. We suggest that the different outcomes of empirical studies revealing elevation patterns in seed size may be a result of a mountain-specific dependence of environmental variables on elevation and differential selection pressures on plants. A clearer picture can be obtained if local ecological factors are thoroughly analyzed and if plant reproductive strategy is viewed more widely, including a seed size vs. seed number trade-off.

## Supporting information

S1 Dataset Dataset used in statistical analysis. (XLSX)

## References

[1] Mazer SJ. Seed mass of Indiana Dune genera and families: taxonomic and ecological correlates. Evol. Ecol. 1990; 4:326–357.

[2] Leishman MR, Wright IJ, Moles AT, Westoby M. The evolutionary ecology of seed size In: Fenner M, editor. Seeds: the ecology of regeneration in plant communities. Oxon: CABI International; 2000 p. 31–57.

[3] Moles AT, Westoby M. Seedling survival and seed size: a synthesis of the literature. J. Ecol. 2004; 92:372–383.

[4] Silvertown J. The paradox of seed size and adaptation. Trends Ecol. Evol. 1989; 4:24–26. doi: 10.1016/0169-5347(89)90013-X 21227308 10.1016/0169-5347(89)90013-X

[5] Simons AM, Johnston MO. Variation in seed traits of *Lobelia inflate* (Campanulaceae): sources and fitness consequences. Am. J. Bot. 2000; 87:124–132. 10636835

[6] Dalling JW, Hubbell SP. Seed size, growth rate and gap microsite conditions as determinants of recruitment success for pioneer species. J. Ecol. 2002; 90:557–568.

[7] Shaukat SS, Siddiqui ZS, Aziz S. Seed size variation and its effects on germination, growth and seedling survival in *Acacia niloticia* subsp. *indica* (Benth.) Brenan. Pakistan J. Bot. 1999; 31:253–263.

[8] Turnbull LA, Paul-Victor C, Schmid B, Purves DW. Growth rates, seed size, and physiology: do small-seeded species really grow faster? Ecology 2008; 89:1352–1363. 18543628 10.1890/07-1531.1

[9] Stearns SC. The Evolution of Life Histories. Oxford: Oxford University Press; 1992.

[10] Smith C, Fretwell SD. The optimal balance between size and number of offspring. Am. Nat. 1974; 108:499–506.

[11] Geritz SAH. Evolutionarily stable seed polymorphism and small-scale spatial variation in seedling density. Am. Nat. 1995; 146:685–707.

[12] Rees M, Westoby M. Game theoretical evolution of seed mass in multi-species ecological models. Oikos 1997; 78:116–126.

[13] Venable DL, Brown JS. The selective interactions of dispersal, dormancy, and seed size as adaptations for reducing risk in variable environments. Am. Nat. 1988; 131:360–384.

[14] Norden N, Daws MI, Antoine C, Gonzalez MA, Garwood NC, Chave J. The relationship between seed mass and mean time to germination for 1037 tree species across five tropical forests. Funct. Ecol. 2009; 23:203–210.

[15] Thompson K. Seeds and seed banks. New Phytol. 1987; 106:23–34.

[16] Reader RJ. Control of seedling emergence by ground cover and seed predation in relation to seed size for some old-field species. J. Ecol. 1993; 81:169–175.

[17] Jakobsson A, Eriksson O. A comparative study of seed number, seed size, seedling size and recruitment in grassland plants. Oikos 2000; 88:494–502.

[18] Coomes DA. Grubb PJ. Colonization, tolerance, competition and seed-size variation within functional groups. Trends Ecol. Evol. 2003; 18:283–291.

[19] Moles AT, Westoby M. Seed size and plant strategy across the whole life cycle. Oikos 2006; 113:91–105.

[20] Czarnoleski M, Olejniczak P, Mikołajczak P, Lembicz M, Kozłowski J. Fungal endophytes protect grass seedlings against herbivory and allow economical seed production. Evol. Ecol. Res. 2010; 12:769–777.

[21] Boulli A, Baaziz M, M’Hirit O. Polymorphism of natural populations of *Pinus halepensis* Mill. in Morocco as revealed by morphological characters. Euphytica 2001; 119:309–316.

[22] Pluess AR, Schütz W, Stöcklin J. Seed weight increases with altitude in the Swiss Alps between related species but not among populations of individual species. Oecologia 2005; 144:55–61. doi: 10.1007/s00442-005-0047-y 15800741 10.1007/s00442-005-0047-y

[23] Bu H, Chen X, Xu X, Liu K, Jia P, Du G. Seed mass and germination in an alpine meadow on the eastern Tsinghai–Tibet plateau. Plant Ecol. 2007; 191:127–149.

[24] Guo H, Mazer SJ, Du G. Geographic variation in seed mass within and among nine species of *Pedicularis* (Orobanchaceae): effects of elevation, plant size and seed number per fruit. J. Ecol. 2010; 98:1232–1242.

[25] Qi W, Bu H, Cornelissen JHC, Zhang C, Guo S, Wang J, et al Untangling interacting mechanisms of seed mass variation with elevation: insights from the comparison of inter-specific and intra-specific studies on eastern Tibetan angiosperm species. Plant Ecol. 2015; 216:283–292.

[26] Schramm BW, Gudowska A, Kapustka F, Labecka AM, Czarnoleski M, Kozłowski J. Automated measurement of ommatidia in the compound eyes of beetles. BioTechniques 2015; 59:99–101. doi: 10.2144/000114316 26260089 10.2144/000114316

[27] IBM SPSS Statistics for Windows, Version 24. IBM Corp. Armonk, NY. 2016.

[28] Körner C. The use of ‘altitude’ in ecological research. Trends Ecol. Evol. 2007; 22:569–574. doi: 10.1016/j.tree.2007.09.006 17988759 10.1016/j.tree.2007.09.006

[29] Wang Y, Wang J, Lai L, Jiang L, Zhuang P, Zhang L, et al Geographic variation in seed traits within and among forty-two species of *Rhododendron* (Ericaceae) on the Tibetan plateau: relationships with altitude, habitat, and phylogeny. Ecol. Evol. 2014; 4:1913–1923. doi: 10.1002/ece3.1067 24963385 10.1002/ece3.1067 PMC4063484

[30] Kichenin E, Wardle DA, Peltzer DA, Morse CW, Freschet GT. Contrasting effects of plant inter- and intraspecific variation on community-level trait measures along an environmental gradient. Funct. Ecol. 2013; 27:1254–1261.

[31] Dolezal J, Dvorsky M, Kopecky M, Liancourt P, Hiiesalu I, Macek M, et al Vegetation dynamics at the upper elevational limit of vascular plants in Himalaya. Sci. Rep. 2016; 6:24881 doi: 10.1038/srep24881 27143226 10.1038/srep24881 PMC4855180

[32] Dori T, Moe SR, Klein JA, Wang S, Totland Ø. Performance of two alpine plant species along environmental gradients in alpine meadow ecosystem in central Tibet. Ecol. Res. 2016; 31:417–26.

[33] Bing H, Wu Y, Zhou J, Sun H, Luo J, Wang J, et al Stoichiometric variation of carbon, nitrogen and phosphorus in soil and its implication for nutrient limitation in alpine ecosystem of Eastern Tibetan Plateau. J. Soils Sediments 2016; 16:405–416.

[34] He X, Hou E, Liu Y, Wen D. Altitudinal patterns and controls of plant and soil nutrient concentrations and stoichiometry in subtropical China. Sci. Rep. 2016; 6:1–9. doi: 10.1038/s41598-016-0001-8 27052367 10.1038/srep24261 PMC4823659

[35] Hofmann K, Lamprecht A, Pauli H, Illmer P. Distribution of prokaryotic abundance and microbial nutrient cycling across a high-alpine altitudinal gradient in the Austrian Central Alps is affected by vegetation, temperature and soil nutrients. Microbial Ecol. 2016; 3:704–716. 10.1007/s00248-016-0803-z 27401822

[36] Hniličková H, Kuklová M, Hnilička F, Kukla J. Effect of altitude and age of stands on physiological response of three dominant plants in forests of the Western Carpathians. Plant, Soil Environ. 2016; 62:341–347.

[37] Eriksson O. Game theory provides no explanation for seed size variation in grasslands. Oecologia 2005; 144:98–105. doi: 10.1007/s00442-005-0001-z 15791431 10.1007/s00442-005-0001-z

